# Understanding the Nuances of Hepatic Portal Venous Gas in Pneumatosis Intestinalis: An Indication of Bowel Ischemia?

**DOI:** 10.7759/cureus.45330

**Published:** 2023-09-15

**Authors:** Saliha Erdem, Suraj V Patel, Dhruvil Patel, Shivam Patel, Shlok Patel, Ahmed Jamal Chaudhary

**Affiliations:** 1 Internal Medicine, Wayne State University School of Medicine, Detroit, USA; 2 Internal Medicine, Ross University School of Medicine, Miramar, USA; 3 Biomedical Sciences, University of South Florida, Tampa, USA; 4 Pharmaceutical Science, University of Michigan, Ann Arbor, USA; 5 Internal Medicine, DMC Sinai-Grace Hospital, Detroit, USA

**Keywords:** bowel ischemia, hpvg, hepatic portal venous gas, pneumatosis cystoides intestinalis, pneumatosis intestinal

## Abstract

Pneumatosis intestinalis (PI) is a relatively rare gastrointestinal finding that has a wide variety of causes - ranging from benign to life-threatening. It is described as the pathological presence of gas within the bowel wall with multiple hypotheses emerging as to the likely mechanism. An important indicator of a life-threatening source of PI is the presence of gas within the hepatic portal vein, referred to as hepatic portal venous gas (HPVG). While non-specific for isolated PI, HPVG has been reported in PI patients to be associated with bowel ischemia and is thereby considered an indication for emergent management. Herein we report a case involving an atypical presentation of altered mental status in which the patient was found to have PI with contemporaneous HPVG. These findings have been reported to have a high mortality rate. Our patient rapidly deteriorated during their hospital course, expiring shortly after being deemed a poor surgical candidate due to their severe co-morbidity burden. Through this case, we review evidence supporting the management of patients with PI and concurrent HPVG from an extensive review of available literature. While PI is a non-specific finding and commonly a source of diagnostic confusion, a better understanding of its natural course and potentially unorthodox sequela may afford more directed and crucial care for critically ill patients, in which time is often a precious commodity.

## Introduction

Pneumatosis intestinalis (PI), also referred to as pneumatosis cystoides intestinalis, intraluminal bowel gas, pneumatosis coli, or intestinal emphysema, is a multifactorial condition characterized by the accumulation of gas in the extraluminal spaces of the small or large intestine [1, 2]. While not well characterized, the pathogenesis of PI has been hypothesized to be linked to mechanical, pulmonary, and bacterial processes, all of which relate to the breakdown of the mucosa by way of increased intra-intestinal pressure [2]. Symptoms are often vague and can consist of abdominal discomfort, loss of appetite, diarrhea, hematochezia, tenesmus, and constipation [3]. Interestingly, the effects of PI can range from benign to lethal with PI estimated to be present in 0.03% of the overall population from an autopsy series, which is likely to be an underestimated figure [4, 5]. Due to this variability, PI lacks a concrete diagnostic profile, as many asymptomatic cases go unreported and patients with vague or inexact symptoms tend to be misdiagnosed, only being discovered upon abdominal imaging evaluation [6, 7]. While the pathogenesis of PI remains elusive, multiple hypotheses put forth in literature attempt to explain the root cause. Three such hypotheses are the: (1) Pulmonary theory, whereby chronic pulmonary disease reduces lung integrity, subsequently leading to the release of emphysematous gas into the mesentery that can localize to the bowel, (2) Mechanical theory, in which injury to the intestines can cause an elevation of intraluminal pressure leading to localized bowel wall gas accumulation and injury, and (3) Bacterial theory, where seeding of gas-producing bacteria into the mesentery affords the opportunity for intramural gas accumulation within the bowel [2].

Treatment options are stratified based on whether the cause of the PI is primary or secondary in nature. Primary PI constitutes 15% of cases, which contain relatively benign findings and are managed with medical treatment and/or outpatient observation [4, 7]. Secondary PI cases (85%) originate as a consequence of other diseases and reflect life-threatening pathological conditions requiring emergent intervention [4, 7]. Currently, there is no unified standard for treatment as primary PI and secondary PI require drastically different forms of intervention and can exhibit a wide range of symptoms [3]. Therefore, the majority of PI interventions focus on treating the associated causative illness [8].

PI can be screened for through radiography, which displays morphological changes in the intestinal wall caused by gas inflammation. A Computed Tomography (CT) scan showing increased thickness of the bowel wall containing gas is considered pathognomonic and is highly suggestive for the diagnosis of PI [4, 9]. While rare, the prognosis of PI is worsened with the presence of hepatic portal venous gas (HPVG), which can give way to portomesenteric gas, a radiological finding indicative of the loss of bowel integrity that allows gas to enter the venous circulation [10]. The presence of HPVG has been associated with partial bowel ischemia with varying outcomes, yet consensus does indicate that it is a sign for surgical intervention [11]. Herein we present a case of PI with extensive HPVG in an elderly woman initially presenting with altered mentation, discussing her presentation, laboratory and radiographic findings, and clinical course.

## Case presentation

A 91-year-old female with a history of metastatic breast cancer in remission since 2021 and essential hypertension was brought to the emergency department by her daughter due to bizarre behavior. Her daughter found the patient to be praying on her hands and knees for twenty hours while only consuming scarce amounts of oatmeal and nutritional drinks during that time. The patient was referred to as being “in a great state of mind” by the daughter during these events, but due to the prolonged nature of this behavior, she was brought in for evaluation. On arrival, the patient had altered mentation and appeared drowsy, but was arousable to touch. She was alert and oriented to two out of four prompts, which was below her baseline per her daughter. Physical examination was notable for a diffusely rigid abdomen with tenderness to palpation and intentional guarding with absent rebound tenderness. Additionally, bilateral shin abrasions were also found, which was likely a consequence of her prolonged kneeling. She was hypotensive on arrival, with a blood pressure of 97/62 mmHg. All other vital signs, including temperature, heart rate, respiration rate, and oxygen saturation were within normal limits.

Initial testing revealed the patient to be hypoglycemic, which when corrected with multiple D50 (dextrose 50%) injections, stabilized her blood glucose levels with no improvement in mentation or abdominal symptoms. She also received a 2-L bolus of Lactated Ringers, which did mildly improve her responsiveness. Both urinalysis and urine drug screening were unremarkable. Labs showed a drastic leukocytosis of 38,500 cells/mm3 and an elevated lactic acid level of 6.6 mMol/L. Furthermore, the labs showed the patient to have hypertransaminasemia with an aspartate aminotransferase (AST) of 925 mg/L and an alanine transaminase (ALT) of 172 mg/L, as well as an elevated alkaline phosphatase (ALP) level of 257 mg/L. There was also a severe elevation of creatine kinase of >60,000 U/L. The serum electrolyte panel demonstrated hyperkalemia, hyponatremia, and an elevated blood urea nitrogen/creatinine ratio suggestive of pre-renal azotemia. Serum troponins levels were 699 ng/L with no endorsing chest pain (Table 1). The patient was immediately started on empiric intravenous ceftriaxone and metronidazole for the potential of an intra-abdominal source of infection as a cause of sepsis. Blood cultures drawn on admission later grew *Escherichia coli* susceptible to ceftriaxone, permitting the continuance of the antibiotic regimen for which the patient remained on for the rest of the admission.

**Table 1:** Electrolytes, BUN, Creatinine, Lactic Acid, Troponin, WBCs, CK lab values on and after hospital admission. Abbreviations: BUN, blood urea nitrogen; AST, aspartate, transaminase; ALT, alanine transaminase; ALP, alkaline phosphatase; CK, creatine kinase; WBC, white blood cells.

Laboratory Tests	Time of Admission	4 hours post-Admission	24 hours post-Admission	Reference Ranges
Sodium (mMol/L)	134	138	141	136-145
Potassium (mMol/L)	5.8	5.3	5.6	3.5-5.1
Chloride (mMol/L)	97	102	112	98-107
Bicarbonate (mMol/L)	13	13	11	21-31
Anion Gap (mMol/L)	24	23	18	4-12
Glucose (mMol/L)	50	305	271	70-100
BUN (mg/dL)	39	42	56	7-25
Creatinine (mg/dL)	2.35	2.29	2.75	0.70-1.30
Magnesium (mg/dL)	-	-	2.5	1.6-3.0
AST (mg/L)	925	1486	-	8-48
ALT (mg/L)	172	360	-	7-55
ALP (mg/L)	257	194	-	44-147
CK (U/L)	>60,000	-	5925	30-145
Lactic Acid (mMol/L)	6.59	4.8	8.3	<2
Troponin (ng/L)	699	545	633	<0.04
WBC (cells/mm^3^)	38,500	23,600	-	3,500-10,500

**Figure 1:**
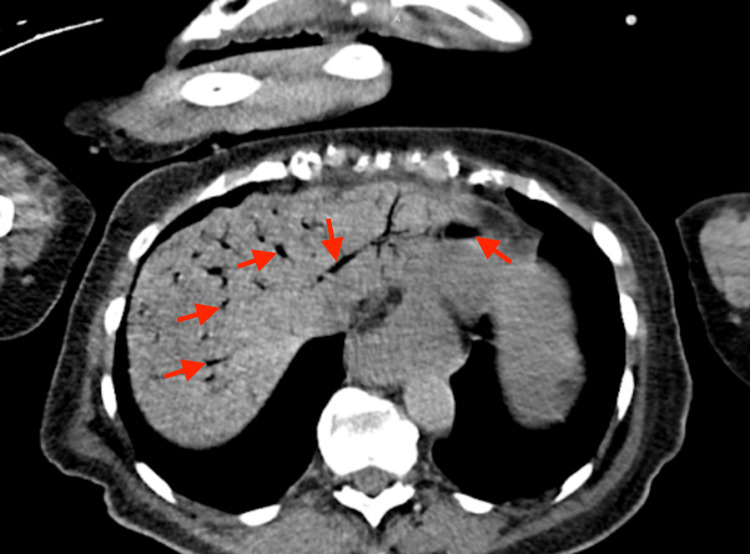
CT Abdomen/Pelvis without contrast (axial view) Diffuse hepatic portal venous gas within the liver (shown by the red arrows).

**Figure 2:**
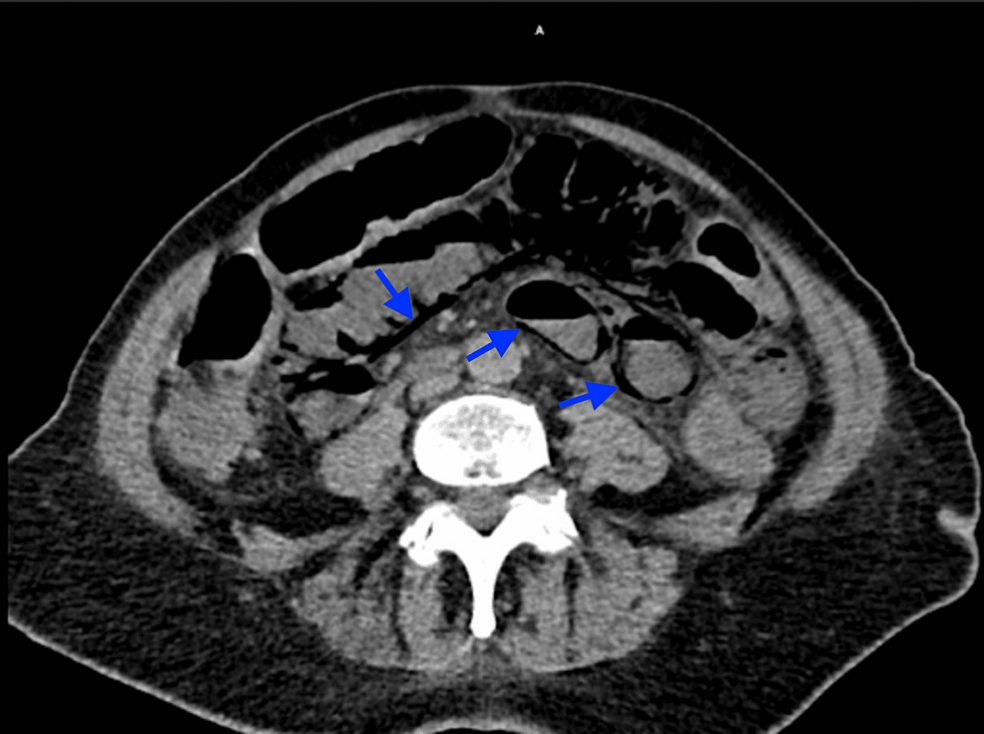
CT Abdomen/Pelvis without contrast (axial view) Pneumatosis intestinalis (shown by the blue arrows) within the proximal portion of the small bowel, the middle portion of the small bowel, and the transverse colon as a consequence of ischemic colitis.

An initial electrocardiogram (ECG) was negative for any acute ischemic pathology of the heart. Due to the patient's continued altered mentation and abdominal symptoms, a non-contrast CT scan of the head and abdomen was performed. The head CT scan demonstrated mild, generalized cortical atrophy and periventricular small vessel chronic ischemia with no acute neurological manifestations. Results of the abdominal CT scan revealed massive HPVG within the liver (Figure 1) with findings indicative of PI involving both the proximal and mid-small bowel with likely progression towards the transverse colon (Figure 2). 

Given these findings, the intra-facility general surgery service was consulted, who deemed the patient to be a poor surgical candidate given her current condition and comorbidities. She was immediately admitted to the medical intensive care unit (MICU) where she was started on vasopressor therapy with norepinephrine. The patient remained hypotensive, despite being on single-pressor support. Of note, she experienced a transient episode of atrial fibrillation with a rapid ventricular rate, which quickly subsided without the need for invention. Ultimately, after eight hours of receiving care in the MICU, the patient expired from a cardiac arrest. No autopsy was done per the family's wishes.

## Discussion

We present a case of PI with concurrent massive HPVG. While PI can have an ominous prognosis, there exists a wide spectrum in terms of severity, ranging from benign with a low risk of hospitalization to severely debilitating with a high mortality. This is due to PI having multiple possible sequelae, ranging from bowel obstruction to bowel ischemia [12, 13]. The incidence of both PI with subsequent HPVG is very rare, with the most likely source identified by cohort studies to be bowel ischemia as per autopsy evaluations [14]. Other sources for HPVG include distal obstruction of the bowel, which increases intraluminal back-pressure and translates that pressure into the bowel walls, and inflammatory pathologies that induce tissue changes that promote air translocation to and from the bowel [12]. 

Yet, in a cohort study by Milone et al., HPVG presence does not have a high confirmatory rate for solely bowel ischemia [15]. HPVG in PI patients is associated with a 44.5% increase in mortality when compared to patients without this finding [16]. The source of the gas in PI is likely from a combination of mechanical and bacterial mechanisms as intestinal barriers are broken down through ischemic changes, which allows aerobic intestinal bacteria to release harmful contents into the venous circulation that pool in the portal vein [17, 18]. This relationship was, importantly, present in our case, which likely was the mechanism contributing to the patient's acute hospital course and mortality.

Of note, HPVG has also been described in select case reports involving lower-risk and transient gastrointestinal (GI) pathologies and procedures [19]. Cases involving patients with Crohn's disease, viral gastroenteritis, and uncomplicated endoscopic procedures have shown HPVG as a concurrent finding, which contrasts with the historical notion of HPVG being exclusively associated with PI in the context of bowel ischemia [19]. The perceived increase in cases of HPVG without PI has been hypothesized to be a result of the increased quality in radiographic imaging and screening for relatively benign GI conditions, leading to an increased detection rate of HPVG in the absence of PI [19].

The most common symptom associated with intestinal ischemia and resultant bowel necrosis is severe abdominal pain. Subsequent diarrhea, vomiting, and abdominal distention are also observed, which coincide with symptoms of PI. There are currently no specific clinical manifestations specific to PI, and therefore patients with these symptoms instead require an abdominal radiographic evaluation. Our patient though presented with altered mentation and a physical examination with non-specific abdominal findings. A presentation of altered mentation in a PI patient is rarely reported [20]. We provide evidence that in such patients, an abdominal examination and radiographic evaluation may prove useful in diagnostic evaluation. Lab values when investigating PI can have a broad range, ranging from within normal limits to grossly abnormal. In the setting of bowel ischemia, as in our patient, thresholds included elevated lactate (>2 mmol/L) and acidotic pH (<7.3) combined with a low bicarbonate level of <20 mEq/L [7]. These features can indicate that necrosed bowel is a likely cause of symptoms and these features were all present in our patient’s initial lab evaluations and persisted throughout her hospitalization course. While our patient had confirmed bowel tenderness, these features also exist in a septic presentation, which informed our initial evaluation for an intra-abdominal infection - including an abdominal radiographic evaluation and empiric antibiotic therapy.

While the initiating event of PI can be from various sources, further complications are from the anaerobic intestinal bacterial activity which induces worsening changes from activity. Therefore antibiotics, specifically 500 mg of metronidazole, and a bowel rest regimen are considered first-line management for PI with resolution of symptoms in a majority of mild- to moderate-severity cases [21]. Conversely, conservative observation treatment also has a very high success rate of resolution but was not considered in our patient due to ischemic bowel findings [22, 23]. The presence of significant bowel ischemia as a cause of PI carries a high indication for surgical intervention, which has a high success rate in curing PI and subsequent sequelae [23]. This was the cause for our surgical consultation, which did not progress forward due to the patient's advanced age and comorbidities. 

Interestingly, a cohort study from Della Seta et al. found that the presence of HPVG in the setting of PI caused by bowel ischemia has a deleterious prognosis, and such patients have minimal benefit from surgical intervention. This is due to the higher likelihood of mortality during the post-surgical period within their cohort [16]. Another proposed alternative therapy is hyperbaric oxygen, working two-fold to lower the partial pressure of gases within the venous system while acting as a toxin to anaerobic bacteria, thus halting their capacity to inflict injury upon the bowel [2].

Given the complex course of our patient and her rapid deterioration, it is difficult to ascertain the definitive cause of the PI and subsequent bowel ischemia. Given our patient’s history and hospital course, alternative theories have been proposed as to explain her condition. These theories include the PI being linked to pulmonary pathologies like chronic obstructive pulmonary disease, chemotherapeutic exposure causing tissue toxicity, and primary cardiovascular risk factors such as atrial fibrillation, which can cause ischemic conditions precipitating primary gastrointestinal tract damage [17, 24]. Our patient had multiple atherosclerotic risk factors and experienced one transient episode of atrial fibrillation during their hospitalization. The patient did not have a history of atrial fibrillation, yet she may have had paroxysmal atrial fibrillation prior to hospital evaluation. Additionally, atherosclerotic, calcified vessels seen in advanced age along with the lack of caloric intake prior to the patient's presentation may have been an inciting event of her bowel changes manifested by PI. Furthermore, there are very few investigations on predictors of mortality from PI. Greenstein et al. found that those with a leukocytosis of >12 cc/m^3^ with an age >60 years old and confirmed PI were likely to undergo surgical intervention with a survival rate upwards of 80% in their cohort [25]. This suggests surgical management to be an ideal course for patients with PI related to bowel ischemia, as in our patient, yet the presence of multiple co-morbidities contraindicated any such intervention.

## Conclusions

In this article, we report a uniquely atypical case of PI with a presentation of altered mental status. In patients with such presentations and abnormalities in their abdominal examination, evaluation through abdominal imaging is crucial in attempting to determine the root cause. The presence of HPVG, while not a highly-specific finding in cases of severe bowel ischemia with PI, is a concerning finding for individuals with PI when found on radiography in terms of prognostication. Therefore, prompt management with antibiotics and immediate surgical evaluation should be performed by care teams to ensure that any emergent source is expeditiously identified and accounted for. In patients who are poor surgical candidates, conservative antibiotic treatment has had success in relieving PI, yet was unsuccessful in our patient, likely due to their unfavorable medical history. Ultimately, caregivers should look to HPVG as a sign to intervene quickly and efficiently when suspecting acute bowel ischemia in patients where PI is found in order to allow for improved patient outcomes.
